# Clinical efficacy and safety of proton radiotherapy for ocular conjunctival malignancies: a systematic review and meta-analysis

**DOI:** 10.3389/fpubh.2025.1486988

**Published:** 2025-02-11

**Authors:** Tingwei Zheng, Dandan Wang, Yuxin Miao, Meng Dong, Qin Liu, Qiuning Zhang, Huiling Bai, Hongtao Luo, Meixuan Li

**Affiliations:** ^1^The First School of Clinical Medicine, Gansu University of Chinese Medicine, Lanzhou, Gansu, China; ^2^Gansu Provincial Hospital, Lanzhou, Gansu, China; ^3^Institute of Modern Physics, Chinese Academy of Sciences, Lanzhou, China; ^4^Gansu Provincial Hospital of TCM, Lanzhou, Gansu, China; ^5^Evidence-based Medicine Center, School of Basic Medical Sciences, Lanzhou University, Lanzhou, Gansu, China

**Keywords:** proton beam therapy, ocular conjunctival malignancies, systematic review, meta-analysis, effectiveness and safety

## Abstract

**Objective:**

The use of proton beam therapy (PBT) for treating ocular conjunctival malignancies is on the rise across numerous medical centers. This study conducts a systematic review and meta-analysis to assess the effectiveness and safety of PBT in treating malignant conjunctival tumors.

**Methods:**

We searched for studies on PBT for ocular conjunctival malignancies in PubMed, Embase, Cochrane Library, and Web of Science (WoS) databases up to November 25, 2023. Studies were selected and data were extracted by two independent reviewers based on pre-established inclusion and exclusion criteria. The quality of evidence was assessed using the GRADE method. Meta-analysis was performed using STATA version 16.0.

**Results:**

An initial search yielded 586 articles, from which six retrospective case series studies were selected involving 291 patients with ocular conjunctival malignancies, including 240 cases of conjunctival melanoma and 51 cases of conjunctival squamous cell carcinoma (SCC). Meta-analysis with a random-effects model showed that PBT is effective and relatively safe, with 2-, 4-, and 5-year overall survival (OS) rates of 98% (95% CI 95–102%), 87% (95% CI 69–104%), and 78% (95% CI 70–87%) respectively. Reported toxicity rates included 19% for cataracts, 10% for glaucoma, 5% for lacrimal stenosis, 52% for sicca symptoms, and 11% for limbal stem cell deficiency. The GRADE assessment yielded a low certainty of evidence.

**Conclusions:**

Proton therapy offers a viable alternative treatment for patients with conjunctival malignancies, with acceptable treatment-related toxicity rates.

## 1 Introduction

Ocular surface malignancies, including conjunctival melanoma (CM) and SCC, affect primarily middle-aged and older adults and pose significant ocular and systemic threats (1, 2). Despite their low prevalence, these tumors are notoriously aggressive, leading to potential blindness or tumor-related mortality, and require intensive treatment (3). Managing unresectable diffuse conjunctival malignancies is challenging; treatments risk damaging essential ocular functions and may lead to vision loss or, in extreme cases, enucleation. Therapeutic efforts to preserve ocular function cover various modalities, such as surgical resection, cryotherapy, immunotherapy, chemotherapy, and radiotherapy (4). In cases of conjunctival SCC, tumors classified as T2–T3 show higher recurrence rates post-surgery (5). Treatments with local chemotherapy and immunotherapy are widely used and effective (MMC 74%; IFN 72–85%; 5 FU 57%). However, issues of secondary complications and recurrence remain significant clinical challenges (6–9).

In recent years, advances in radiation therapy such as PBT have introduced significant innovations. The development of more compact and cost-effective single-chamber proton beam devices has facilitated the widespread adoption of this technology (10). PBT utilizes an external beam where radiation dosage is precisely controlled. Unlike conventional radiotherapy, PBT delivers a lower incident dose and concentrates the majority of radiation directly on the tumor, reducing exposure to surrounding healthy tissues (11). Produced via a linear accelerator or cyclotron, the proton beam reaches the tumor with high energy and forms a narrow, intense Bragg peak. Beyond this peak, the energy falls off sharply, allowing precise energy deposition at the target. This dosimetric property provides a clear advantage over conventional radiation therapy, enhancing treatment effectiveness while protecting nearby normal tissue. During planning, the physician carefully defines the tumor target at the Bragg peak's apex to optimize tumor destruction and minimize damage to adjacent structures (12). Studies suggest that proton radiotherapy's radiobiological effects may offer a stronger tumoricidal effect than photon radiotherapy, potentially improving treatment outcomes (13, 14). Thus, PBT represents a promising approach for treating conjunctival tumors. Although clinical studies of PBT have been limited to small samples and case reports, the efficacy and safety are not fully established. Therefore, this study aims to systematically review and analyze the evidence on PBT's effectiveness in treating conjunctival tumors to support clinical treatment, guideline development, and policy implementation for PBT.

## 2 Materials and methods

This systematic review and meta-analysis was registered with PROSPERO (registration number CRD42023486574) and adhered to the PRISMA guidelines (15) and additional methodological standards (16–18).

### 2.1 Search strategy

A comprehensive search was conducted in the Cochrane Library, Embase, PubMed, and WoS databases for articles published through November 25, 2023. Only English publications were included. The search terms used were: ((((((conjunctival lymphoma) OR (conjunctival melanoma)) OR (Conjunctiva)) OR (squamous cell conjunctival cancer)) OR (conjunctival cancer)) OR (conjunctival neoplasms)) AND (((((proton radiotherapy) OR (proton radiation therapy)) OR (proton radiation)) OR (proton irradiation)) OR (proton therapy)). Additionally, references from relevant reviews and primary studies were examined to identify further pertinent articles.

### 2.2 Inclusion and exclusion criteria

Inclusion criteria:

(a) Population: Patients diagnosed with conjunctival malignancy;
(b) Intervention: PBT alone or in combination with other therapies;
(c) Comparison: No restrictions;
(d) Outcomes: OS, toxicity;
(e) Study type: No restrictions.

Exclusion criteria:

(a) Use of radiotherapy modalities other than PBT;
(b) Duplicate study reports;
(c) Case reports, reviews, meta-analyses, abstracts, letters, commentaries, and consensus statements;
(d) Studies lacking sufficient data;
(e) Studies on irrelevant topics.

### 2.3 Study selection

All articles were processed in EndNote 20 to eliminate duplicates. Titles and abstracts were independently screened by two reviewers to exclude irrelevant studies, and the full texts of potentially relevant studies were assessed for eligibility. Discrepancies were resolved through discussion or by consulting a third reviewer. Data extraction included:

(a) First author, year, research institution, country, study design, and period;
(b) Patient demographics, tumor specifics, total treatment dose, and follow-up;
(c) Outcomes focusing on survival rates and toxicity;
(d) Risk of bias assessment items.

### 2.4 Risk of bias assessments

To assess the quality of the case series studies, a scale developed by the Canadian Institute of Health Economics (IHE) was used (19). This method comprises 20 items distributed over seven areas, each assessed as “yes”, “no”, or “unclear”. A study was considered to have acceptable quality and moderate risk of bias if more than 14 items received a “yes” rating. Disagreements were resolved through discussion among researchers or consultation with a third party.

### 2.5 Statistical analysis

Descriptive statistics were used to summarize basic characteristics and toxicity incidence. Dichotomous data were presented as frequencies and percentages, and continuous data were summarized as means or medians with their respective ranges. Meta-analysis employed a random-effects model in STATA 16.0, calculating effect sizes as proportions with 95% confidence intervals (CI). The *I*^2^ statistic assessed heterogeneity, with *I*^2^ ≤ 50% and *P* ≥ 0.05 indicating homogeneity; values outside this range suggested heterogeneity. Publication bias was evaluated using a funnel plot for outcomes from at least ten studies.

## 3 Results

### 3.1 Study selection and characteristics

**Figure 1:**
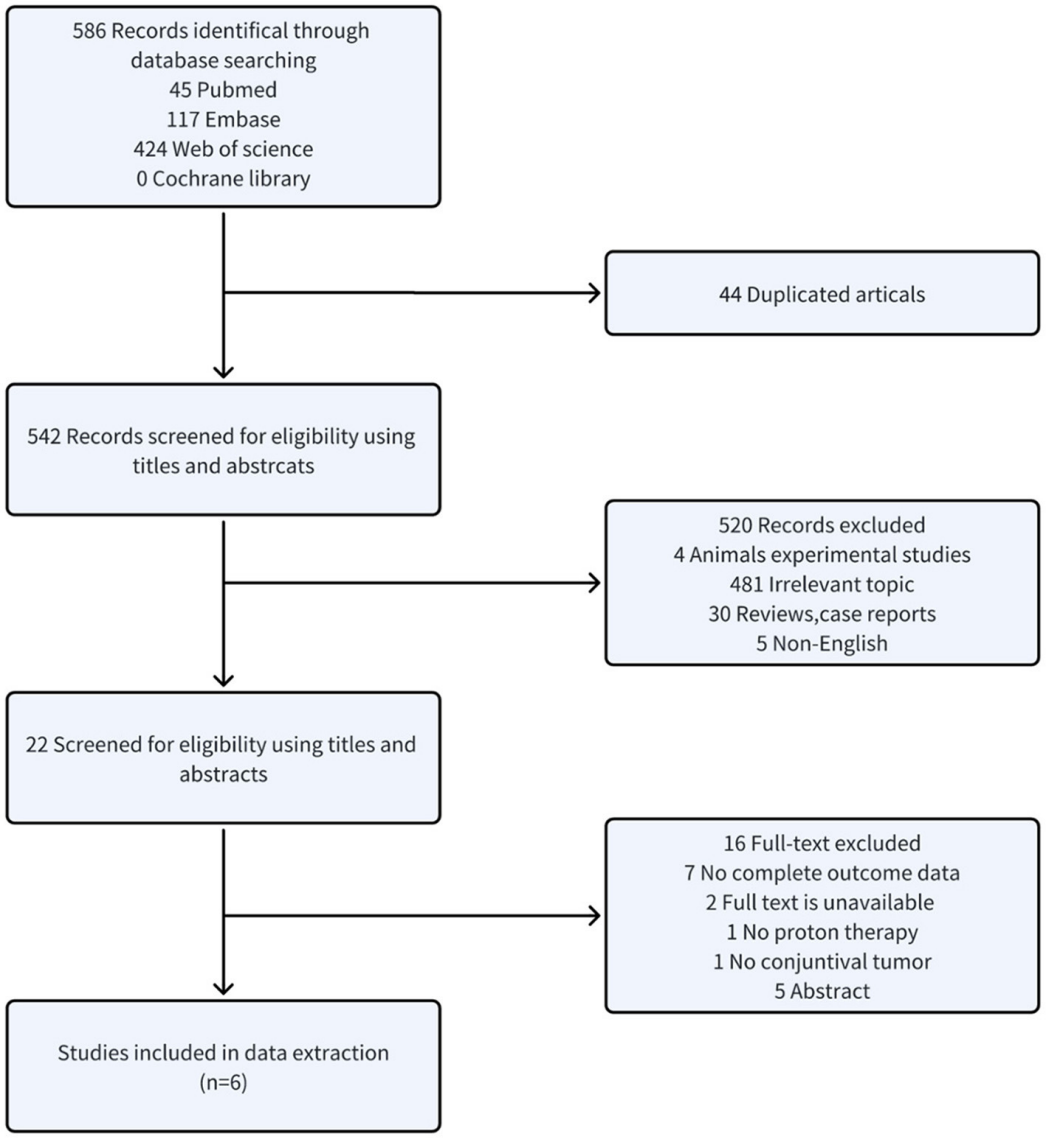
Flow diagram.

As illustrated in Figure 1, the search yielded 586 studies. After removing 44 duplicates, 542 studies remained for title and abstract screening. Of these, 522 were excluded as unsuitable. The full texts of the remaining 22 studies were examined; 16 were excluded for reasons including seven with incomplete data, two lacking full text, one missing proton beam irradiation, one lacking conjunctival malignancies, and five that were abstracts. Ultimately, six retrospective studies involving 291 patients were included.

### 3.2 Basic characteristics

Six retrospective case series studies were included, involving 291 patients with conjunctival tumors treated with proton therapy (20–25). Of these, four studies focused on CM (*n* = 240) (20, 22–24), and two addressed conjunctival SCC (*n* = 51) (21, 25). The studies originated from Germany (published in 2006 and 2019) (22, 23), France (published in 2013 and 2019) (20, 21, 24), and Italy (published in 2022) (25). The sample size ranged from 15 to 92 patients, with follow-up periods spanning 24 to 50.4 months (Table 1).

**Table 1:** Basic characteristics of included studies.

**Study**	**Country**	**Study design**	**Age (years)**	**follow-up (months)**	**Research year range**	**No.of Patients**	**Male/female**	**TNM stage**	**Dose (GyE/Fr)**
Wuestemeyer et al. (22)	Germany	retrospective	65(65 ± 16)	34	1993–2002	20	9/11	NA	45Gy/2 or31Gy/6
Maschi-Cayla et al. (20)	France	retrospective	63 (33-92)	33	1992.01–2012.01	39	NA	T1: 32 T2: 6 T3: 1	36Gy/6 or16Gy/2
Santoni et al. (21)	France	retrospective	71	24	2002–2017	54	37/16	miSCC: T1:6(42.9%) T2:2(14.2%) T3:6(42.9%) SCC: T1:9(23.1%) T2:7(17.9%) T3:23(59.0%)	45Gy/8
Scholz et al. (23)	Germany	retrospective	66.2	50.4	1993.09–2015.05	89	52/37	T1: 5 T2: 49 T3: 35 PAM: 53 Non-PAM: 33	45Gy/8
Thariat et al. (24)	France	retrospective	63	32.4	1992–2018	92	51/41	T1: 63 (71.6%) T2: 13 (14.8%) T3: 12 (13.6%)	45Gy/8
Milazzotto et al. (25)	Italy	retrospective	61 (31–87)	48	2010–2019	15	11/4	NA	48–60Gy/4

### 3.3 Outcomes

The articles covered conjunctival malignant melanoma and squamous cell tumors. Three studies reported on metastasis-free survival (20, 22, 23), four on OS (20, 21, 23, 25), and two on local relapse-free survival (20, 23). Additionally, one study reported on PFS (24), and another on DFS (25) (Table 2).

**Table 2:** The main results of the included studies.

**Study**	**Reported Findings**	**Local recurrence and DM**	**Metastasis**	**Deaths**	**Toxicity**
Wuestemeyer et al. (22)	Metastasis-free survival: 3 y: 57%	6/20	6 (30%)	4	Symptoms in the treated eye: 19 A limbal stem cell deficiency with corneal vascularisation: 4 Segmental cataract: 7 (35%);
Maschi-Cayla et al. (20)	OS: 5y: 81% Metastasis-free survival: 5y: 91% Local relapse-free survival: 5y: 69%	In the first 3 months after initial surgery: 3 (14%) Remaining patients: 8 (47%)	4 (10%)	2	NA
Santoni et al. (21)	OS: 2y: 98.9% OS: 5y: 83.4% Incidence of local relapse: 2y: 14.8%	miSCC:4/15 SCC:4/39	6	10 (18.5%)	Irritative symptoms: 20 (37.0%) Cataract in 14 (38.89%) Lid alopecia: 11 (30.6%) Eyelid dermatitis: 5 (13.9%) Neovascular glaucoma: 3 (8.3%) Cutaneous retraction: 2 (5.6%) Lacrimal duct stenosis: 2 (5.6%) Retinal vein occlusion: 2 (5.6%) Basal cell carcinoma of the inner eyelid: 1
Scholz et al. (23)	OS: 5y: 71% Metastasis-free survival:5y: T2: 71%; T3: 71% PAM:71%; nonPAM:72% Local relapse-free survival: 5y: T2: 45%; T3: 51% PAM: 47%; non-PAM:51%	29/89	14 (16%)	*NA*	Limbal stem cell deficiency: 7 (8%) Secondary glaucoma: 10 (11%) artificial tear drops: 27 (30%) cataract: 15 (19%)
Thariat et al. (24)	PFS: 2y: 84.2% 5y: 61.5%	25/90	NA	*NA*	Cataract: 22 (23.9%) Glaucoma: 13 (14.1%) Conjunctival, corneal thinning, and scleral perforation: 9, 11, and 1 Conjunctival scarring: 7 (7.6%) Madarosis: 21 (22.8%) Lacrymal duct stenosis and dry eye syndrome: 5 (5.5%) and 28 (30.4%) Macular edema: 1 various other mild complications: 17
Milazzotto et al. (25)	OS: 4y: 86.6%; DFS: 4y: 86.6%	2/15	NA	2 (not related to the disease)	Eyelid oedema:1 Cataract: 1 Conjunctivitis: 1 Conjunctivitis, red-eye syndrome: 1 Eyelid perforation: 1 Lacrimal duct stenosis: 1

### 3.4 Risk of bias and certainty of evidence assessment results

**Figure 2:**
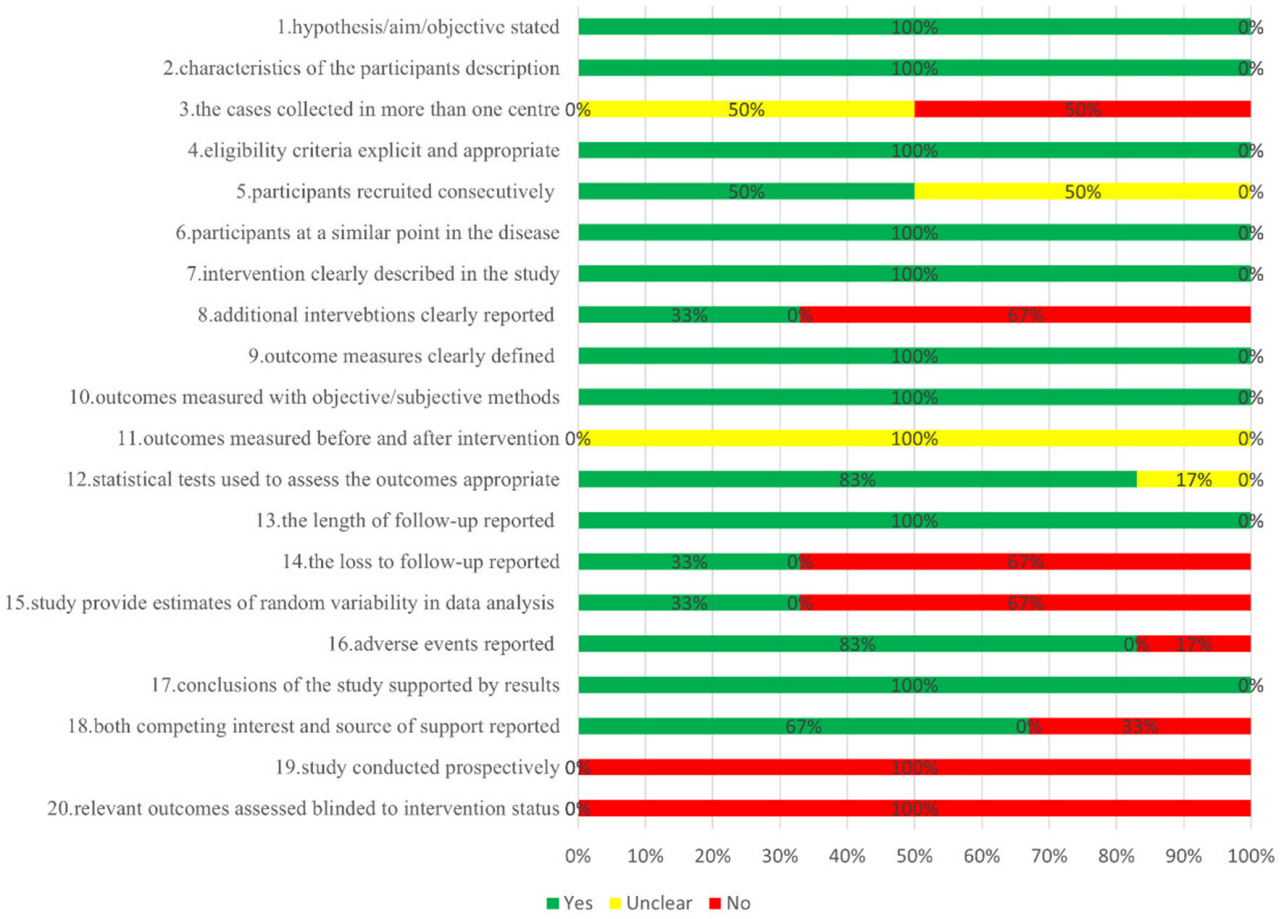
Risk of bias assessment of included case series study of proton therapy for ocular conjunctival malignancies.

Figure 2 depicts the overall suboptimal quality of the included case series, primarily due to their retrospective nature and the lack of blinding among outcome assessors. Three studies were conducted at a single center (21, 23, 24); three used consecutive participant recruitment (21, 22, 24); none measured outcomes before and after intervention; two reported patient loss to follow-up (20, 22); two provided estimates of random variables in data analyses of relevant outcomes (20, 21). GRADE profiler was used to assess the quality of evidence for proton radiotherapy's effects over 2, 4, and 5 years and its impact on cataracts, glaucoma, lacrymal duct stenosis, limbal stem cell deficiency, and sicca symptoms. The evidence for OS and the ocular conditions is considered LOW certainty, while the evidence linking to sicca symptoms is rated VERY LOW (Table 3).

**Table 3:** Grading of the quality of evidence in case series studies of proton therapy for conjunctival malignancies.

**Outcomes**	Illustrative comparative risks^*^(95% CI); assumed risk corresponding risk	**Relative effect (95% CI)**	**No of participants**	**Quality of the evidence (GRADE)**
OS = 2 years	Low 0 per 1,000; 0 per 1,000	OR 0.98 (0.95 to 1.02)	54 (1 study)	low^1, 2, 3, 4, 5^
OS = 4 years	Low	RR 0.87 (0.69 to 1.04)	15 (1 study)	low^a, b, c, d, e^
OS = 5 years	Low	RR 0.78 (0.70 to 0.87)	182 (3 studies)	low ^a, b, c, d, e^
Cataract	Low	OR 0.19 (0.12 to 0.26)	270 (5 studies)	low ^a, b, c, d, e^
Glaucoma	Low	RR 0.10 (0.05 to 0.15)	235 (3 studies)	low ^a, b, c, d, e^
Lacrymal duct stenosis	Low	RR 0.05 (0.01 to 0.08)	161 (3 studies)	low ^a, b, c, d, e^
Sicca symptoms	Low	RR 0.52 (0.10 to 0.94)	201 (3 studies)	very low ^a, b, c, d^
Limbal stem cell deficiency	Low	RR 0.11 (0.01 to 0.21)	109 (2 studies)	low ^a, b, c, d, e^

^a^There is a risk of uncertainty bias; ^b^There is a risk of high bias; ^c^The sample size is small; ^d^Many clinical applications; ^e^according to I^2^, the statistical heterogeneity was small. ^*^The basis for the assumed risk (e.g., the median control group risk across studies).

### 3.5 OS

**Figure 3:**
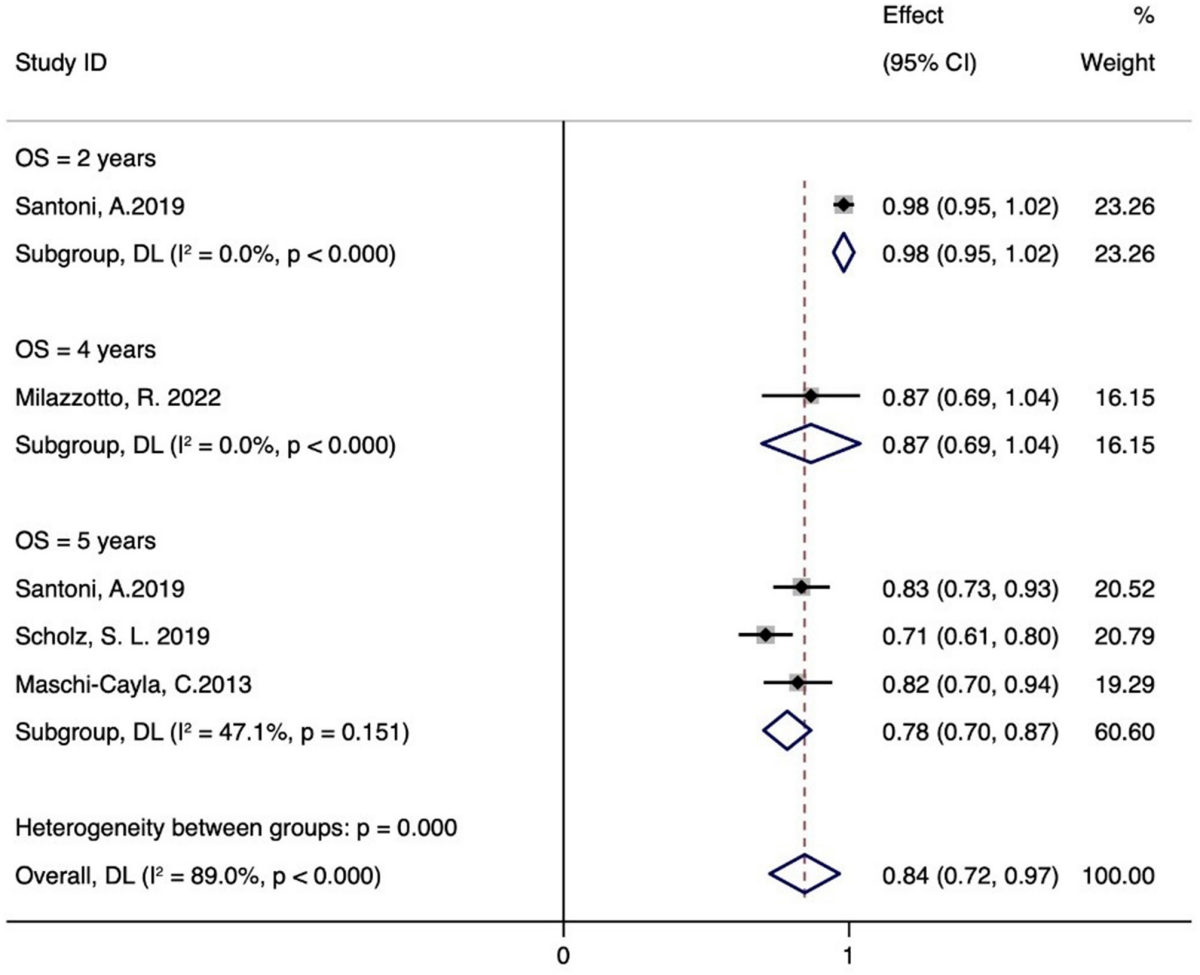
OS rate of proton therapy for ocular conjunctival malignancies.

Figure 3 illustrates six case series studies on PBT for ocular conjunctival malignancies. These include reports on 2-year OS (21), 4-year OS (25), and 5-year OS (20, 21, 23); one study did not include OS data (22). Five studies analyzed the OS associated with PBT (20, 21, 23, 25). A random-effects meta-analysis showed the 2-, 4-, and 5-year OS rates to be 98% (95% CI 95-102%), 87% (95% CI 69-104%), and 78% (95% CI 70–87%), respectively.

### 3.6 Toxicity

**Figure 4:**
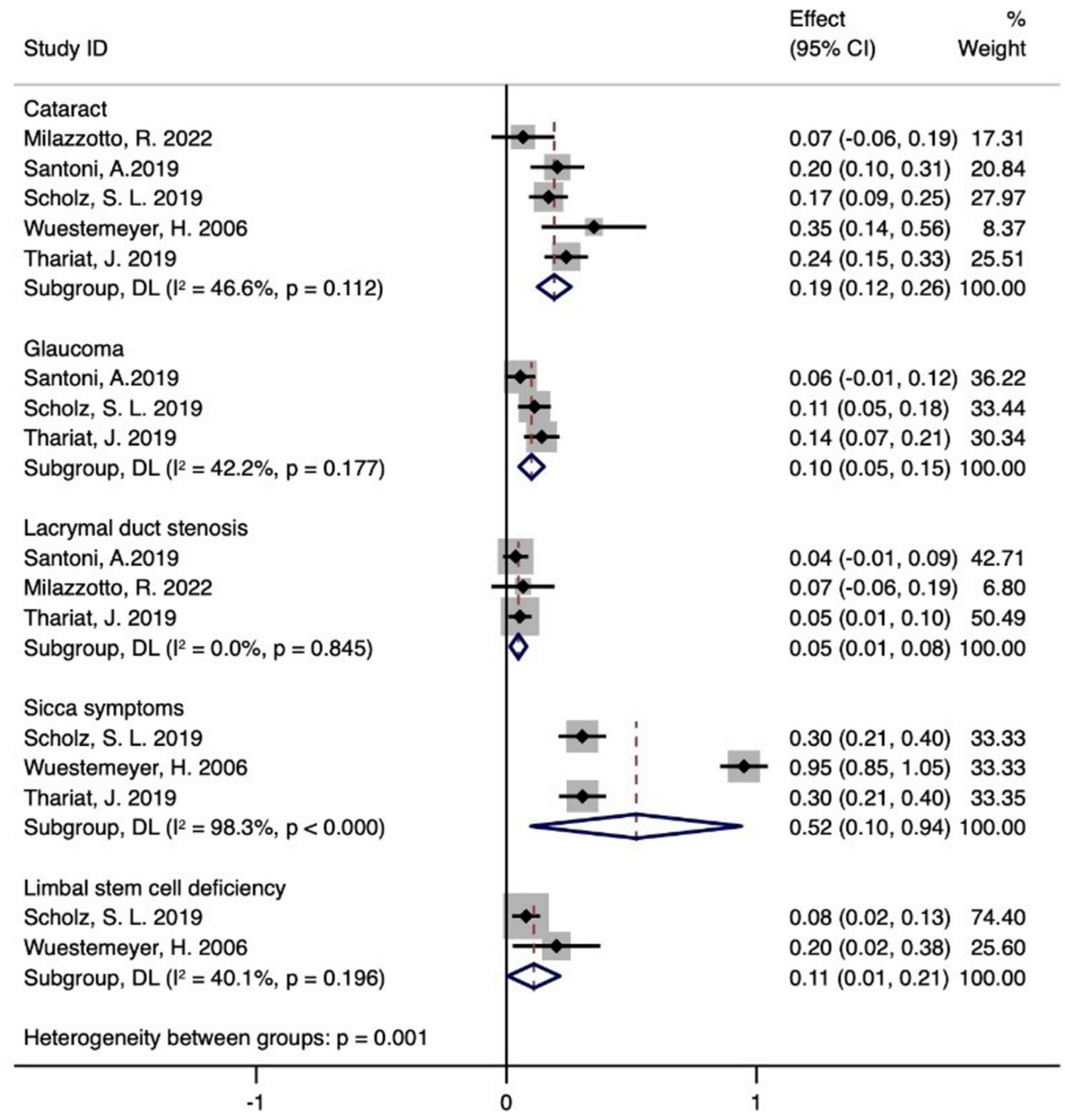
Toxicity of proton therapy for ocular conjunctival malignancies.

Figure 4 shows that five studies documented adverse effects (21–25), with five reporting cataracts (*n* = 59) (21–25), three detailing glaucoma (*n* = 26) (21, 23, 24), and three describing lacrimal duct stenosis (*n* = 8) (21, 24, 25). Three studies reported sicca symptoms (*n* = 51) (22–24), and two noted limbal stem cell defects (*n* = 11) (22, 23). The meta-analysis indicated the following incidence rates for proton therapy: 19% for cataracts (95% CI 12–26%), 10% for glaucoma (95% CI 5–15%), 5% for lacrimal duct stenosis (95% CI 1–8%), 52% for sicca symptoms (95% CI 10–94%), and 11% for limbal stem cell defects (95% CI 1–21%). Additional adverse reactions included eyelid alopecia (30.6%, *n* = 11), eyelid dermatitis (13.9%, *n* = 5), and skin degeneration (5.6%, *n* = 2) reported by Santoni et al. (21). Retinal vein occlusion (5.6%, *n* = 2) and inner eyelid basal cell carcinoma (*n* = 1) were also reported. Thariat et al. noted conjunctival thinning, corneal thinning, scleral perforation (*n* = 9, 11, and 1, respectively), conjunctival scarring (*n* = 7), osteoporosis (*n* = 21), macular edema (*n* = 1), and other minor complications (*n* = 17) (24). Milazzotto et al. reported eyelid edema, cataract, conjunctivitis, red-eye syndrome, eyelid perforation, and lacrimal duct stenosis (all *n* = 1) (25).

## 4 Discussion

We analyzed six peer-reviewed studies involving 291 patients from Germany, France, and Italy. The results suggest that PBT for conjunctival malignancies offers high survival rates and an acceptable level of treatment-related adverse effects. The quality of the methodologies in the studies was considered modest, and the confidence in the evidence ranged from very low to low.

Conjunctival malignancies, though rare, pose significant challenges in ophthalmology. These tumors often display aggressive behavior, requiring intensive treatments that may lead to severe ocular complications, including blindness, and potentially result in tumor-related mortality. The primary conjunctival malignancies include SCC, malignant melanoma, and malignant lymphoma, with surgical treatment being predominant for SCC and malignant melanoma. Often, adjuvant treatments such as local chemotherapy or radiotherapy are necessary (3). A comprehensive therapeutic approach is adopted, involving surgical excision, brachytherapy, cryotherapy, chemotherapy, systemic immunotherapy, photon radiation therapy, and PBT, tailored to the tumor's characteristics and the patient's health.

An independent study of 31 patients with CM reported a survival rate of 75.8% at 2 years and 51.5% at 5 years (26). Another study reported a five-year disease-free survival (DFS) rate of 67.5% for patients with conjunctival lymphoma treated with brachytherapy (27). Surgical interventions have shown a five-year recurrence rate of 36% to 45%, highlighting the variability and challenges in long-term effectiveness (28–33). Our meta-analysis revealed that PBT achieved a 98% two-year and a 78% five-year OS rate. These findings suggest that the five-year survival rate with proton therapy may exceed those of surgical methods. Additionally, a study found an 86.6% four-year DFS rate after PBT for conjunctival SCC (25), indicating potential advantages over brachytherapy. Our analysis also showed a five-year recurrence rate of 31% in patients treated with PBT for malignant melanoma (20), demonstrating its potential superiority over surgical methods (34). Scholz et al. (23) noted a 16% incidence of metastasis after PBT, highlighting its effectiveness in controlling metastasis compared to cryotherapy. However, the limited empirical evidence and the retrospective nature of the studies may affect the reliability of these findings.

PBT has emerged as a promising and revolutionary treatment modality within radiation oncology. Its precision targeting, enhanced by the Bragg peak phenomenon, significantly improves therapeutic efficacy. This advanced approach not only aims to reduce the adverse effects associated with conventional radiotherapy but also expands the range of treatable conditions, providing new management options for various oncological diseases. In managing conjunctival malignancies, adverse reactions are an inevitable challenge. It is notable that many patients develop ocular complications post-treatment, underscoring the need for effective management and mitigation of these side effects. One study involving 51 participants who underwent adjuvant electron beam radiation therapy (EBRT) post-surgical excision of CM found that a majority, 96% (25/26), suffered from keratoconjunctivitis sicca, while 27% (7/26) developed localized cataracts, and 15% (4/26) experienced limbal stem cell insufficiency (22, 35, 36). Another study with 24 subjects undergoing postoperative radiotherapy reported a 62% incidence of cataracts (37). Systemic immunotherapy can cause a spectrum of side effects, including fatigue, diarrhea, colitis, hepatitis, pneumonia, and endocrinopathies, along with less common complications. For conjunctival malignancies, chemotherapy is typically used as an ancillary treatment in conjunction with surgery or radiotherapy to enhance efficacy. Although effective in destroying cancer cells, chemotherapy also harms healthy cells, leading to various side effects.

Our meta-analysis found sicca symptoms in 52% of cases, cataracts in 19%, limbal stem cell deficiency in 11%, glaucoma in 10%, and lacrimal duct stenosis in 5%. Due to the high radiation doses required for treating malignant melanoma and SCC, proton therapy offers precise dosing that covers the entire conjunctival region while minimizing exposure to other ocular structures (38). Milazzotto and colleaguesand (21) reported a superior OS rate, possibly due to higher radiation doses. This suggests that proton therapy, either alone or in combination with complex surgical techniques, could be a viable and safe treatment option for this cancer type. The minimal toxicity of proton therapy, due to its dosimetric advantage where radiation dose distribution is more uniform, helps preserve more ocular tissue by avoiding electron scatter. Consequently, the incidence of harmful reactions such as xerophthalmia, cataractogenesis, and limbal stem cell insufficiency is significantly reduced in patients treated with proton therapy for conjunctival malignancies. Our findings show that proton therapy maintains a commendably benign toxicity profile for treating conjunctival malignancies. Managing and controlling adverse reactions after therapeutic interventions in patients with conjunctival malignant tumors is a critical area that requires further research and academic focus.

The quality assessment of the included studies identifies several areas for enhancement in future clinical trials: First, it is crucial to involve multiple centers in patient recruitment to improve the generalizability of the results. Second, comprehensive reporting of any additional interventions is essential. Third, it is imperative to ensure the mandatory disclosure of conflicts of interest and funding sources (16, 39, 40). Finally, the importance of blinding patients in clinical trials must be emphasized (41, 42). Addressing these aspects can significantly reduce the risk of bias in study outcomes (43). The literature analyzed shows a variable risk of bias, often uncertain or high, and is characterized by small sample sizes, necessitating a downgrade in the evidence grading. However, the presence of numerous clinical applications and minimal statistical heterogeneity warrants an upgrade and careful evaluation. The findings suggest that the evidence level for OS at 2, 4, and 5 years, as well as for Cataract, Glaucoma, Lacrymal duct stenosis, and Limbal stem cell deficiency, is generally rated as LOW. The evidence level for Sicca symptoms is rated as VERY LOW, which calls into question the reliability of the current results. The overall level of evidence is deemed average, highlighting the need for future studies to include multi-center, high-quality randomized controlled trials for further investigation.

Although systematic reviews and meta-analyses suggest that PBT is an effective treatment for conjunctival malignancies, these findings should be cautiously interpreted and applied due to notable limitations. The variability in clinical efficacy observed among patients could stem from the lower quality and small number of studies in this meta-analysis, highlighting the need for additional research to explore this observation. Moreover, despite a thorough literature search and stringent inclusion criteria, only six studies met the inclusion standards, potentially impacting the robustness of the findings. Furthermore, the predominance of retrospective studies in the analysis curtails the scope for more exhaustive comparative studies. While PBT is emerging as a promising clinical treatment option, its growing acceptance underscores the imperative for more extensive prospective studies to validate its efficacy.

## 5 Conclusions

Proton therapy holds promise as a viable alternative treatment for patients with conjunctival malignancies, with acceptable levels of treatment-related toxicity. However, the confirmation of PBT's superiority over other radiotherapy modalities necessitates further high-quality prospective randomized controlled trials.

## Data Availability

The original contributions presented in the study are included in the article/supplementary material, further inquiries can be directed to the corresponding authors.
